# Delirium education priorities for healthcare professional students: a modified Delphi study

**DOI:** 10.1186/s12909-025-07667-w

**Published:** 2025-07-29

**Authors:** Tara Anderson, Alice Coffey, James McMahon, Stephanie Craig, Heather E. Barry, Margaret Graham, Jill Murphy, Christine Brown Wilson, Pauline Boland, Dympna Tuohy, Matt Birch, Audrey Tierney, Patrick Stark, Arlene McCurtin, Laura Creighton, Elizabeth Henderson, Lana Cook, Emma Cunningham, Geoffrey M. Curran, Gary Mitchell

**Affiliations:** 1 School of Nursing and Midwifery, Queen’s University Belfast, Belfast, UK; 2 School of Nursing and Midwifery, University of Limerick, Limerick, Ireland; 3 School of Pharmacy, Queen’s University Belfast, Belfast, UK; 4 School of Allied Health, University of Limerick, Limerick, Ireland; 5 School of Medicine, Dentistry and Biomedical Sciences, Queen’s University Belfast, Belfast, UK; 6 Center for Implementation Research, University of Arkansas for Medical Sciences, Little Rock, AR USA

**Keywords:** Delirium; Education; Education, Professional; Students, Health Occupations; Interdisciplinary Studies; Medicine; Nursing; Allied Health Occupations; Delphi Technique; Health

## Abstract

**Background:**

Delirium is frequently unrecognised, and healthcare professionals lack knowledge and confidence in recognition and management of the condition. Increased delirium education within pre-registration healthcare profession curricula may help to address this. A modified Delphi approach was utilised to develop a set of education priorities associated with delirium education for healthcare profession students.

**Methods:**

An initial list of 72 education priorities were identified from a literature review, stakeholder focus groups, and a review of available clinical guidelines. Priorities were grouped into eight domains ‘assessment and diagnosis’, ‘aetiology and risk factors’, ‘pathophysiology’, ‘treatment of delirium’, ‘prevention’, ‘delirium and dementia’, ‘impact on people with delirium, informal caregivers and family’ and ‘health promotion and public education’. Academic experts and healthcare professionals were invited to rank each priority and each domain across two rounds. Round one consisted of a survey including the list of 72 potential education priorities which participants were asked to rank from one (not a priority) to five (essential priority). Items which did not reach consensus criteria following round one were re-distributed as round two of the survey and participants were asked to repeat this exercise for the shorter list of items. Additionally, in round two, participants were asked to rank the eight domains from most important to least important.

**Results:**

Eighty participants completed round one of the Delphi survey and 55 (68.75%) also completed round two. Following round one, 41 priorities were considered highly relevant and therefore met the criteria to be included in the final set of education priorities. An additional four items reached these criteria following round two and so the final set consists of 45 items. Priorities related to prevention, diagnosis, and treatment were consistently ranked highly whereas priorities related to pathophysiology and health promotion were consistently ranked lowest.

**Discussion:**

This Delphi study identified areas of education viewed as the highest priorities for healthcare profession students’ delirium education, drawn from a range of academics and healthcare professionals. The final set may help to inform the development of delirium education reflecting these priorities.

**Supplementary Information:**

The online version contains supplementary material available at 10.1186/s12909-025-07667-w.

## Background

Delirium is characterised by an acute change in attention and awareness, accompanied by disturbances in cognition (such as memory, orientation, language, and perception), which develops over a short period of time [1]. These changes cannot be explained by a pre-existing neurocognitive disorder [1, 2]. Additionally, a delirium diagnosis requires evidence that the disturbance is a physiological consequence of another medical condition such as infection, dehydration, or substance intoxication [1]. Prevalence rates vary depending on the patient population as delirium is relatively uncommon in outpatient settings but is estimated to affect almost 20% of medical inpatients over the age of 75 years, and over 50% of mechanically ventilated patients [2, 3]. The condition is preventable [4], but frequently underdiagnosed [5].

A lack of confidence and knowledge may explain the under-recognition of delirium. For example, nurses have reported a lack of confidence leading to frustration with the assessment of delirium [6]. In another study, nurses have been found to lack knowledge of the diagnostic criteria of delirium, and have difficulty distinguishing between delirium, dementia, and depression [7, 8]. Uncertainty around recognition and management of delirium has also been found within junior doctors [9, 10] and allied health professionals including occupational therapists, speech and language therapists, and physiotherapists [11]. These findings highlight knowledge gaps at multiple stages of delirium care, including both prevention and management. Additionally, hypoactive delirium is especially prone to under-recognition due the subtlety of its clinical features [12]. It is also evident that knowledge gaps are present across a wide range of healthcare professions including nurses, doctors, and allied health professionals.

This lack of delirium knowledge across healthcare professionals is especially concerning due to the interprofessional nature of the delivery of delirium care. Collaboration is viewed as a key factor in enhancing delirium care, and therefore, interprofessional educational initiatives have been highlighted as a potential method for improving the identification and management of delirium [13]. Although evidence is limited, interprofessional education within delirium care may positively influence patient outcomes [14] and is recommended in line with recent NICE guidelines which state interventions should be delivered by a multidisciplinary team trained in delirium prevention [15]. A lack of delirium knowledge, and interprofessional education, may lead to delayed or missed diagnosis, and incomplete or inappropriate interventions, which may contribute to prolonged episodes of delirium, increased rick of complications, and poorer patient outcomes [16, 17].

It is therefore evident that increased delirium education is needed across health professions. Recent evidence has shown the effectiveness of delirium education in practice, leading to increased knowledge, and improved patient outcomes [18]. For example, a cross-professional facilitated delirium group objective structured clinical examination education intervention was suggested to be effective in increasing delirium knowledge, communication, and clinical reasoning for medical students [19]. Delirium education within the pre-registration nursing curriculum may also increase knowledge and confidence [20, 21]. Although several studies suggest increased delirium education may lead to improvements in knowledge, recognition, and confidence in delirium care [15–19], improvements in patient outcomes have not been consistently observed or reported across studies. Therefore, while delirium education is a promising strategy, further research is needed to explore its impact on patient outcomes. Additionally, effective delirium education may be enhanced from reflecting the needs of those working in delirium care. For example, family involvement and education, and non-pharmacological management have been identified as training priorities to enhance delirium care among intensive care unit (ICU) and ward nurses [22].

The present study aims to further identify and develop education priorities within delirium care for health profession students, utilising a modified Delphi approach. This is an iterative method of gathering the opinions of experts in the field by which group consensus may be achieved [23]. This process enables the education priorities to be ranked as of greater or lower importance to ensure those of greater importance are reflected within delirium education. This Delphi study is part of a wider project which aims to co-design an interdisciplinary, all-Ireland digital education resource to improve the prevention, recognition, and management of delirium among pre-registration healthcare profession students. The primary goals of the resource are to enhance students’ (1) knowledge, (2) confidence, and (3) interprofessional collaboration related to delirium care. These outcomes will be evaluated using pre- and post-intervention questionnaires. Additionally, we plan to conduct follow-up focus groups with students in the months after receiving the education to explore how, and if, the learning has translated into their clinical practice experiences [24]. This project consists of three phases: (1) a review of the literature [25], (2) attain consensus relating to delirium education through a Delphi study and co-design with stakeholders, and (3) acceptability testing of the resource. This paper focuses on the Delphi study conducted as part of phase two. This Delphi study aimed to achieve consensus on education priorities using a Delphi survey completed by a panel of experts which will then be used to inform the development of the digital education resource about delirium for healthcare profession students.

## Methods

### Design

A modified Delphi technique was employed to develop a consensus on education priorities associated with delirium education for healthcare profession students. The Delphi method is a validated and systematic approach for achieving consensus within the health and social sciences [23, 26], which involves the distribution of surveys to an anonymous panel of experts. Survey distribution is conducted in rounds with participant responses to a previous round informing the next.

The present study utilised a modified Delphi process which consisted of two rounds between December 2023 and April 2024. An initial first round of qualitative, open-ended questions, typical of a Delphi method, was not included within this study as the aim was to refine education priorities previously identified within a systematic review of the literature [25]. Although conventional Delphi methods may include more rounds, it was determined a priori that this modified Delphi process would consist of two rounds to minimise attrition and participant burden. The reporting of this study conforms to the DELPHISTAR reporting guidelines [27]. The Delphi study was conducted by an interdisciplinary team with representatives from nursing, medicine, pharmacy, public health, occupational health, health promotion and implementation science.

### Recruitment/ population

Both academic experts and healthcare professionals, from the island of Ireland, were invited to participate in the study. Experts were defined as individuals with substantial experience in delirium care, either through clinical practice or academic research. Healthcare professionals were required to confirm that they worked regularly with individuals at high risk of or experiencing delirium, such as in intensive care units, acute surgical wards, fracture clinics, or dementia care settings. Academic experts were defined as researchers who were actively publishing in the field of delirium care. All potential participants who met these criteria were invited to complete the first round of this Delphi study, and all those who completed the first round were invited to participate in the subsequent round. Recruitment was supported by the Royal College of Nursing’s (RCN) Older People Forum, the British Geriatrics Society, the Irish Gerontological Society, and the NICE Clinical Guideline Committee for CG103 (Delirium: Prevention, Diagnosis and Management). These networks consist of both clinical and academic experts within care for older people and/or delirium care.

Potential participants were emailed a study invitation letter and participant information sheet by the relevant organisation (e.g. RCN). Within a week of receiving this documentation, potential participants were then emailed to confirm whether they wished to participate. Both Delphi surveys were developed and completed online via Microsoft Forms, into which consent forms were incorporated. Participants were asked to complete each survey within a three-week period of receiving the link.

### Development of the Delphi survey

Education priorities were identified from a previous literature review [25], stakeholder focus-group interviews [28], and a review of available clinical guidelines (including the Health Service Executive Ireland Delirium guidance, Delirium Hub Education and Training Resources from the British Geriatrics Society, NICE guidelines on Delirium: Prevention, Diagnosis and Management, Scottish Intercollegiate Guidelines Network (SIGN), European Delirium Association, American Delirium Society, and Australasian Delirium Association) which were compiled to develop a draft list of 106 items which may be viewed in the Supplementary Material. Prior to round one of the Delphi study, the original 106 evidence-informed education priorities, derived from a systematic review, stakeholder consultation, and clinical practice guidelines, underwent a pre-consensus screening process. This process was conducted by the research team in collaboration with an expert reference group established for the wider project. This group comprised two medical delirium specialists, two delirium nurse specialists, two delirium researchers, and one individual with lived experience of delirium, in addition to all co-authors listed on this manuscript. The selection criteria for this step included ensuring clarity of language, removing overlapping or duplicate items, and excluding those judged to be out of scope or not actionable within the context of undergraduate curricula. Items were removed only through group consensus following discussion. This step ensured the relevance and face validity of the items selected for Delphi consideration.

To facilitate the Delphi process and ease interpretation of the priorities, items were grouped into eight relevant domains: assessment and diagnosis; aetiology and risk factors, pathophysiology; treatment of delirium; prevention; delirium and dementia; impact on people with delirium, informal caregivers and family; health promotion and public education. These domains were developed by the research team, drawing on thematic analysis from the literature review, stakeholder interviews, and clinical guidelines. This approach was taken by design, as no existing interdisciplinary pre-registration healthcare education framework for delirium was identified in the literature to guide domain development.

The full list of potential priorities may be viewed in the Supplementary Material. Participants were asked to prioritise each item using a five-point Likert scale ranging from one (not a priority) to five (essential priority).

#### First round

The first round of the Delphi survey was distributed in December 2023. Round one included a demographic question (‘Please indicate your job roles or expertise in delirium care’) to allow presentation of the range of expertise within the participant sample. This was followed by the 72-item list which participants were asked to prioritise. This list of items is presented in the Supplementary Material.

#### Second round

**Fig. 1:**
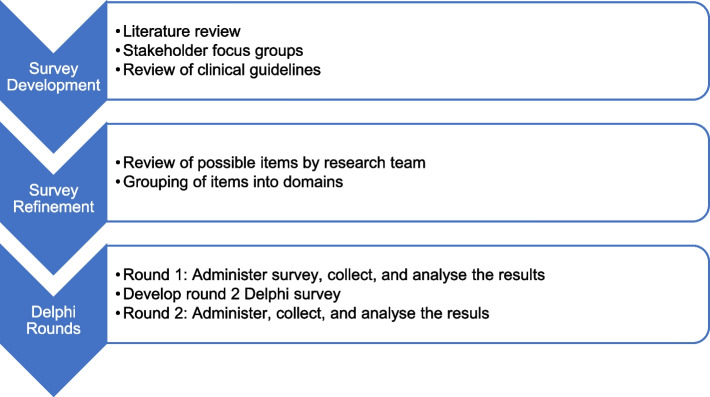
Process of the modified Delphi study

The second round of the Delphi survey was distributed in February 2024. Based on the results of the first round, the list of items was reduced, and participants were asked to complete the same exercise of prioritising this shorter list of items. The round two survey, therefore, presented only the items which did not reach consensus criteria following round one. However, participants were also shown the full list of original items from round one, with clear indication of which items had already achieved consensus and which had not. This was done to ensure participants had full visibility of the overall landscape of education items under consideration and could contextualise their responses accordingly. The full list of items presented in round two may be viewed in the Supplementary Material as those marked “included”. In addition, a ranking question was included following the prioritisation exercise. This asked participants to rank the eight domains from one (most important) to eight (least important). The ranking exercise in the second round of the Delphi survey served as a means to corroborate the prioritised items by ensuring that the items aligned with the participants' prioritised domains, such that if a domain, like prevention, was ranked as a high priority but no prevention-related items were included in the prioritised list, it indicated an inconsistency that needed further review. Figure 1 presents an overview of the modified Delphi study process.

### Ethics

Queen’s University Belfast, Faculty of Medicine, Health and Life Sciences Research Ethics Committee granted ethical approval for this study (Ref: MHLS23_140) after considering benefits and risks and ensuring participants’ autonomy would be respected. All participants in this study were provided with an information sheet outlining the purpose, procedures, and potential risks involved in each stage of the Delphi process. They were given the opportunity to read this information thoroughly and ask any questions prior to participation. Each participant signed an online consent form for each stage of the Delphi study, indicating their informed consent to participate. No identifiable information was collected within the surveys. Once responses were downloaded from Microsoft Forms to Excel, the Forms data was deleted. All methods were performed in accordance with the Declaration of Helsinki [29].

### Analysis

Descriptive analysis using Microsoft Excel was undertaken for responses to both the first and second Delphi rounds. Responses from each participant were weighted equally. The consensus threshold was set at 75%, as is commonly cited within Delphi studies [30], and items were categorised as highly relevant (“in”), not relevant (“out”), or no consensus achieved. Therefore, to be considered “in”, at least 75% of participants had to have rated the item as either a high or essential priority (≥ 4) and less than 15% of participants had to have rated the item as either a low priority or not a priority (≤ 2). Items were considered “out” if 75% or more participants rated them as either a low priority or not a priority (≤ 2) and less than 15% of participants rated them as either a high or essential priority (≥ 4). All other items, those not fitting either criterion, were categorised as no consensus achieved.

Items which fell into the no consensus achieved category following round one were re-distributed as round two of the survey. Responses to round two were analysed in the same manner, with the additional items reaching consensus “in” added to those identified in round one. Finally, the ranking exercise was analysed descriptively by calculating the percentage of participants who selected each domain as their first to eighth choice.

## Results

Eighty participants responded to round one of the Delphi survey to develop a consensus on a set of education priorities associated with delirium education for healthcare profession students. Most participants were from a nursing background (*N* = 29), with representation also from medicine, occupational therapy, pharmacy, and research as presented in Table 1. Fifty-five of these participants completed the second round.

**Table 1:** Participant demographic details

Profession	n (%)	Sex (M/F)	Geographical Location (NI/ROI)
Nursing	29 (36.25%)	3 / 26	15 / 14
Medicine	23 (28.75%)	10 / 13	15 / 8
Pharmacy	8 (10%)	4 / 4	6 / 2
Occupational Therapy	8 (10%)	2 / 6	4 / 4
Physiotherapy	2 (2.5%)	0 / 2	1 / 1
Speech & Language Therapy	1 (1.25%)	0 / 1	0 / 1
Research	9 (11.25%)	2 / 7	6 / 3

Following round one, 41 items were considered highly relevant and 31 were categorised as no consensus achieved. No items were excluded following round one as all items either fell within the consensus “in” or no consensus achieved categories. The 31 items which fell into the no consensus achieved category were re-distributed as round two of the survey, following which four of these reached the highly relevant criteria. Again, no items were considered “out” as the remaining 27 items fell within the no consensus achieved category.

A percentage consensus “in” was calculated for each domain of the survey following each round. Following round one, ‘treatment of delirium’ showed the highest percentage consensus “in” (76.9%) while ‘pathophysiology’ was the only domain for which no items met the consensus “in” criteria (0%). Following round two, no change in consensus was found for ‘treatment of delirium’ and so ‘prevention’ became the domain with the highest percentage consensus “in” (80%). ‘Pathophysiology’ remained the domain with the lowest consensus “in” (25%). Results are presented below (Table 2) for the number of items reaching the consensus “in” threshold for each survey domain.

**Table 2:** Number of items reaching consensus “in” for each domain after two Delphi rounds

Survey domain	Total number of items	Number of items reaching consensus “in” (%)	
		**Round 1 (*N* = 80)**	**Round 2 (*N* = 55)**
Assessment and diagnosis	14	9 (64.29%)	9 (64.29%)
Aetiology and risk factors	8	6 (75%)	6 (75%)
Pathophysiology	4	0 (0%)	1 (25%)
Treatment of delirium	13	10 (76.92%)	10 (76.92%)
Prevention	10	7 (70%)	8 (80%)
Delirium and dementia	7	3 (42.86%)	3 (42.86%)
Impact on people with delirium, informal caregivers and family	7	4 (57.14%)	5 (71.43%)
Health promotion and public education	9	2 (22.22%)	3 (33.33%)

### Ranking exercise

In the ranking exercise at the end of round two, 55 participants were asked to rank eight domains related to delirium, from most important (1st) to least important (8th). The total number of rankings collected was 440, with each participant ranking all eight domains once.

The results indicate that ‘treatment of delirium’ was the most highly prioritised domain, with the largest number of participants selecting it as their first choice (32.73%) and consistently ranking it highly across the other positions. ‘Assessment and diagnosis’ was also highly prioritised, with 25.45% of participants ranking it first and many placing it in the second or third ranks. ‘Prevention’ similarly ranked highly, receiving 21.82% of first-place votes and strong support across the ranks. These domains also showed high numbers of items reaching consensus “in” within the prioritisation exercise.

Domains such as ‘aetiology and risk factors’ and ‘delirium and dementia’ received moderate support, with rankings spread across the middle positions. For ‘aetiology and risk factors’, 7.27% of participants ranked it first, while others placed it in the fourth and fifth ranks. ‘Delirium and dementia’ was ranked lower overall, with only 3.64% of participants placing it first, though it received some support in the middle ranks. ‘Impact on people with delirium, informal caregivers, and family’ was similarly ranked in the middle, with mixed responses throughout the ranks.

The domains ranked most important differ slightly to those which showed the highest number of items reaching consensus “in” within the previous exercise. For example, ‘Prevention’ showed the highest number of items reaching consensus “in” whereas, ‘Treatment of delirium’ was most often ranked highest. Additionally, ‘Impact on people with delirium, informal caregivers and family’ was often ranked lower, this domain showed a high number of items reaching consensus “in”.

The domains of ‘health promotion and public education’ and ‘pathophysiology’ received the lowest prioritisation, in line with the previous prioritisation exercise in which the fewest items of these domains reached consensus “in”. ‘Health promotion and public education’ garnered 3.64% of first-place votes and was ranked in the middle to lower positions. ‘Pathophysiology’ was ranked the lowest, with no participants selecting it as their first priority, and it consistently received minimal support across all ranks. The distribution of responses across each rank and domain are shown in Table 3.

**Table 3:** Distribution of ranks for each domain

Rank	Treatment of delirium	Assess-ment and diagnosis	Preven-tion	Aetio-logy and risk factors	Delirium and dementia	Impact on people with delirium, informal caregivers, and family	Health promotion and public education	Patho-physiology
1st	32.73%	25.45%	21.82%	7.27%	3.64%	3.64%	3.64%	1.82%
2nd	18.18%	20%	25.45%	10.91%	9.09%	7.27%	5.45%	3.64%
3rd	14.55%	12.73%	16.36%	12.73%	12.73%	14.55%	10.91%	5.45%
4th	10.91%	9.09%	12.73%	14.55%	16.36%	18.18%	10.91%	7.27%
5th	7.27%	10.91%	10.91%	10.91%	14.55%	16.36%	16.36%	12.73%
6th	5.45%	9.09%	7.27%	16.36%	18.18%	12.73%	20%	10.91%
7th	7.27%	5.45%	10.91%	12.73%	12.73%	14.55%	16.36%	12.73%
8th	3.64%	7.27%	9.09%	14.55%	12.73%	12.73%	10.91%	18.18%

### Final list of educational priorities

The final list of items which were ranked as the highest priority for inclusion within delirium education for healthcare profession students consisted of 45-items. The full list of items is presented, in order of domain ranking, below in Table 4.

**Table 4:** Final list of education priorities

**Treatment of delirium**	
1	Promotion of effective communication and reorientation strategies
2	Highlighting the role of non-pharmacological interventions in delirium treatment
3	Highlighting the role of pharmacological interventions for delirium treatment
4	Education on the judicious use of antipsychotic medications in managing delirium
5	Multidisciplinary team collaboration in tailoring treatment and intervention plans
6	Using evidence-based guidelines for management of delirium appropriate to clinical setting
7	Education on the principles of titration in delirium medication management
8	De-escalation and support for persons in distress
9	Involving the person and caregivers in treatment decisions
10	Fostering a person-centred approach to delirium treatment
**Assessment and diagnosis**	
11	Recognition of the clinical signs and symptoms of delirium
12	Differentiation between hyperactive, hypoactive, and mixed types of delirium
13	Differentiation between delirium and dementia
14	Recording comprehensive history alongside clinical examination
15	Evaluation of delirium fluctuation in symptoms to tailor interventions
16	Application of standardised delirium assessment tools
17	Assessment of delirium risk factors
18	Assessment of delirium in people with communication challenges
19	Education of people, families, and caregivers about recognising early signs of delirium
**Prevention**	
20	Developing strategies for delirium prevention in various settings
21	Creating anticipatory care plans to minimise delirium occurrence
22	Minimising the impact of environmental changes in the prevention of delirium
23	Awareness of the importance of reorientation for people at higher risk of delirium
24	Minimisation of physiological risk factors through pharmacological interventions to reduce delirium risk
25	Highlighting the importance of regular medication review in delirium prevention
26	Promotion of evidence-based communication strategies to minimise delirium occurrence
27	Promoting multidisciplinary care in dementia prevention
**Aetiology and risk factors**	
28	Identification of common risk factors for delirium
29	Understanding the importance of pain in delirium development
30	Recognition of perioperative care as a risk factor in delirium development
31	Recognition of medications associated with delirium risk
32	Analysis of environmental factors influencing delirium development
33	Understanding of the role of infection in delirium
**Delirium and dementia**	
34	Understanding that delirium can occur alongside dementia
35	Awareness of delirium superimposed on dementia (DSD)
36	Highlighting risk factors that predispose individuals with dementia to delirium
**Impact on people with delirium, informal caregivers and family**	
37	Assessing the psychological impact of delirium on the person, their caregivers and family
38	Providing ongoing education to families and caregivers affected by delirium
39	Addressing the emotional impact of delirium
40	Proactive communication with caregivers and families to help them support those affected by delirium
41	Promoting person-centred care in delirium management
**Health promotion and public education**	
42	Prioritising health promotion and public education focused on delirium prevention and recognition
43	Prioritising family and caregiver education on delirium
44	Developing user-friendly educational materials for the public
**Pathophysiology**	
45	Awareness of the underlying pathophysiology of delirium

## Discussion

This Delphi study facilitated the identification of key education priorities regarding delirium for inclusion in an educational resource aimed at healthcare profession students [25]. The findings provide a unique and valuable perspective on delirium education priorities, drawn from the collective expertise of academics and healthcare professionals across a range of disciplines. The final set of education priorities includes 45 items, which encompass all eight proposed domains of delirium knowledge. These domains are: ‘assessment and diagnosis’, ‘aetiology and risk factors’, ‘pathophysiology’, ‘treatment’, ‘prevention’, ‘delirium and dementia’, ‘impact on people with delirium, caregivers and family’, and ‘health promotion and public education’.

The inclusion of items from all eight domains highlights the broad scope of knowledge required for comprehensive delirium education. However, following the two rounds of the Delphi survey, the domains ‘prevention’ and ‘treatment of delirium’ had the highest percentage of items meeting the inclusion criteria for the final set of education priorities. This outcome likely reflects the ongoing challenge of delirium being frequently underdiagnosed [5]. Despite evidence suggesting that increased knowledge of how to prevent and treat delirium could lead to reduced incidence and better patient outcomes [14, 31]. While some research has shown positive effects on staff knowledge and confidence [19, 21], the evidence for consistent improvements in patient outcomes remains mixed [14, 18]. Consequently, these domains are particularly significant for delirium education, as they address gaps in knowledge that may help reduce diagnostic uncertainty and improve management practices among junior doctors, nurses and allied health professionals [7–11].

By contrast, the domains ‘health promotion and public education’ and ‘pathophysiology’ had the lowest percentage of items included in the final set and were consistently ranked lowest in the domain ranking exercise. While these are undoubtedly important areas of delirium education, their lower prioritisation may reflect participants’ preference for domains with immediate clinical applicability. This preference is evident in the higher prioritisation of domains such as ‘treatment’, ‘prevention’, and ‘assessment and diagnosis’. These patterns should be considered when interpreting the final priorities, as they have been influenced by the clinical background and experiences of participants. For example, healthcare professionals often focus on education that directly supports patient care and outcomes, particularly in settings with limited time for in-depth training and this was also acknowledged by healthcare professional students [28]. Consequently, areas such as public education and biological processes may be perceived as less urgent within the context of practical management.

The prioritisation of prevention, recognition, and management of delirium aligns with the focus of existing delirium education initiatives, as evidenced by systematic reviews of the literature [25, 31]. For instance, one systematic review identified 26 studies evaluating educational interventions specifically targeting the recognition of delirium [31]. Among healthcare profession students, research has emphasised the value of experiential learning, including simulated scenarios in which students either experience symptoms of delirium themselves or provide care to simulated patients with delirium [32, 33]. Other studies have assessed the effectiveness of education focusing on the diagnosis and management of delirium [21, 34–36].

The domains ‘delirium and dementia’ and ‘impact on people with delirium, caregivers and family’ received moderate support throughout the Delphi exercises, potentially due to their perceived indirect impact on immediate patient care. Nonetheless, previous research has prioritised the need for further education on differentiating delirium, dementia, and delirium superimposed on dementia [8, 37, 38]. Recognising the impact of delirium on patients, caregivers, and families has also been proposed as an approach to improving patient outcomes [39–41]. The lower levels of prioritisation of these areas in this study may reflect the practical constraints of healthcare education leading to those of a clinical and academic background to favour content directly linked to clinical care over broader topics. Additionally, participants of this study may have prioritised the basics of delirium care for all patients, rather than prioritising the significant subgroup of dementia patients. This highlights the importance of incorporating the views of wide range of stakeholders within the design of delirium education to include healthcare professionals, academics, those with lived experience and the end user to ensure potentially varying priorities of these different groups are considered.

### Impact on education

The identified priorities have important implications for both pre-registration students, and ongoing professional education in healthcare. For example, these priorities may be applicable to training delivered within healthcare settings, such as induction programmes, and continuing professional development. Embedding these priorities across all areas of education for healthcare professionals and students may help to ensure healthcare professionals at all career stages are equipped with the knowledge and skills to recognise, prevent, and manage delirium effectively. Implementation of these priorities will require collaboration between academic institutions, and healthcare organisations who can play a key role in integrating these priorities into education via teaching resources. For example, the findings from this Delphi study will be used within the wider study conducted by the authors to co-design and evaluate a digital educational resource on delirium for health profession students [24].

### Strengths and limitations

This Delphi study benefits from a sample size of 80 first round responses and 55 s round responses. Previous literature suggests varying minimum sample sizes which may range from 10 to 80 participants depending on the homogeneity and expertise of participants [42–44]. The majority of participants were from a nursing background, which may have influenced the prioritisation of items towards a nursing perspective presenting a limitation of this study. Participants within the present Delphi study were, however, from a range of specialties including nursing, medicine, allied health, and research but were all working in a delirium-related field either clinically or academically. All participants had five or more years of experience working in delirium care either through direct clinical care, research, or education, and were based in diverse care settings including hospitals and nursing homes across the Republic of Ireland and Northern Ireland. Therefore, this sample size, at the higher end of recommendations, may be considered a strength of this Delphi study.

Agreement between participants was high with the majority of items reaching consensus after two rounds. This high level of agreement was evident from round one as the final list of 45 items is made up by a majority of items which reached consensus “in” criteria following the first round. Participant attrition between Delphi rounds was relatively high with only 68.75% of participants completing both rounds and therefore, the views of those who did not complete the second round cannot be accounted for. This is a common issue within Delphi studies due to the separate rounds [44–46]. However, the impacts of participant attrition may be limited by the fact the majority of the final set of education priorities within this Delphi study was agreed upon following the first round.

While the use of a modified-Delphi approach, which omitted an open-ended first round, was chosen given this study’s aim to refine education priorities, this may have limited the opportunity for novel insights to emerge directly from participants as the initial list of items was pre-determined. Although this eased participant burden and increased efficiency, this limited the potential for new perspectives. Further, we did not collect specific demographic details such as participants’ exact age, duration of experience beyond the minimum threshold of five years, or geographical data outside of Ireland, which limits the ability to fully characterize the sample and assess the broader representativeness of the expert panel. Additionally, the panel did not include patients, caregivers, or members of the public. While this is a limitation, we were constrained in who we could reach at this stage of the multiphase study, particularly as no dedicated delirium patient and public involvement networks or charities exist within the Irish context. Future phases of the study may seek to include these voices to complement the professional perspective. Moreover, the panel were not able to recruit participants with formal roles in tertiary or workplace education. Including more diverse perspectives may have altered the prioritisation outcomes and should be considered in future research.

Finally, analysis was conducted using Microsoft Excel due to the descriptive nature of the analysis. This presents a limitation to this study as Excel does not generate a script-based record of computational operations and therefore reduces the traceability of data handling. Future studies may enhance methodological rigour by using reproducible, code-based tool for analysis.

## Conclusions

This Delphi study has identified areas of education viewed as the highest priority for healthcare profession students’ education regarding delirium care, drawn from a range of academics and healthcare professionals. Results suggest that education focusing on prevention, diagnosis and management of delirium are of the highest priority while areas related to public education and the biological processes of the condition may be seen as less urgent. The final set of education priorities will help to inform the development of future delirium education for pre-registration healthcare profession students, as well as ongoing professional development training. These findings are particular relevant for curriculum developers, university educators, and healthcare organisations responsible for designing and delivering education to future and current healthcare professionals. To support translation into practice, the prioritised content may be used to guide curriculum design, inform interdisciplinary teaching sessions, or structure simulation-based learning activities that reflect clinical scenarios. The relevance and practicality of future delirium education may be enhanced by aligning content with the identified priorities, and in turn lead to improved prevention, recognition and management of delirium. The original education items were based on a 2023 systematic review and stakeholder interviews and have been checked against current clinical guidance and published literature as of June 2025 to ensure ongoing relevance.

## Supplementary Information

Supplementary Material 1.

## Data Availability

The datasets used and/or analysed during the current study are available from the corresponding author on reasonable request.
