# Supplementary material for: Delirium education priorities for healthcare professional students: a modified Delphi study
Source: BMC Med Educ. 2025 Jul 29;25:1126. doi: 10.1186/s12909-025-07667-w (PMC12308938; doi:10.1186/s12909-025-07667-w)
Supplement: Supplementary file 1 — Supplementary Material 1. [file 12909_2025_7667_MOESM1_ESM.docx]

Supplementary Material – DelHPIre Modified Delphi Study

**Supplementary material 1 – Original List of 106 Potential Delphi Survey Items**

**Diagnosis and Assessment**

1. Recognise the clinical signs and symptoms of delirium, considering its rapid onset.
2. Identify delirium across different patient populations, including older adults, post-operative patients, and paediatric populations.
3. Differentiate between delirium and other cognitive disorders like dementia, emphasising distinctive features.
4. Evaluate delirium severity and fluctuations in symptoms to tailor interventions.
5. Utilise standardised delirium assessment tools effectively for accurate diagnosis.
6. Assess delirium risk factors, including severe illness, medication use, infections, and surgery.
7. Interpret laboratory and imaging findings relevant to delirium diagnosis, promoting diagnostic accuracy.
8. Recognise delirium in patients with comorbid conditions or substance use, considering complex presentations.
9. Evaluate delirium in patients with communication challenges or language barriers, emphasising alternative assessment methods.
10. Consider cultural factors when assessing delirium in diverse patient populations, ensuring cultural competence.
11. Develop comprehensive approaches for delirium diagnosis and assessment, considering interdisciplinary collaboration and diverse settings.
12. Engage in health promotion and public health education to raise awareness about delirium among patients and the community.
13. Educate patients, families, and caregivers about recognising early signs of delirium and the importance of seeking timely medical attention.

**Aetiology and Risk Factors**

1. Identify common risk factors for delirium, such as prolonged hospitalisation and medication-related factors.
2. Recognise medications associated with delirium risk, particularly in older adults, and understand alternatives.
3. Analyse environmental factors influencing delirium development, like changes in surroundings and interventions to mitigate them.
4. Understand the role of infections, including urinary tract infections, in delirium and strategies for prevention.
5. Explore unique risk factors in specific populations, such as paediatric or intensive care patients.
6. Assess the impact of pre-existing cognitive impairment on delirium risk and tailor interventions accordingly.
7. Recognise the role of sleep disturbances in delirium, considering day-night variations and promoting sleep hygiene.
8. Promote interdisciplinary research into the aetiology and risk factors of delirium, fostering innovative preventive strategies.

**Pathophysiology**

1. Explain the underlying pathophysiology of delirium, including neuroinflammation and neurotransmitter imbalances.
2. Describe neurochemical imbalances involved in delirium and their impact on cognitive function.
3. Understand neurodegenerative processes contributing to delirium, especially in older populations.
4. Recognise how delirium accelerates cognitive decline and its long-term effects.
5. Explain the mechanisms of delirium in various patient groups, including those with neurodegenerative diseases.
6. Investigate interdisciplinary research on the pathophysiological aspects of delirium, promoting evidence-based interventions.
7. Comprehend the impact of delirium prevention and management on public health, emphasising cost-effective strategies.
8. Advocate for community-based initiatives to raise awareness about delirium and its prevention among the general population.

**Treatment of Delirium**

1. Prioritise the development of competencies in evidence-based pharmacological interventions for delirium treatment.
2. Educate healthcare providers on the judicious use of antipsychotic medications in managing delirium.
3. Highlight the role of non-pharmacological interventions, including environmental modifications and behavioural strategies, in delirium treatment.
4. Promote the integration of psychological interventions, such as cognitive-behavioural therapy, in the treatment of delirium.
5. Educate healthcare professionals on the potential adverse effects of medications used in delirium treatment.
6. Emphasise the importance of assessing and addressing the underlying causes of delirium during treatment.
7. Prioritise the use of delirium assessment tools in monitoring treatment effectiveness.
8. Promote collaboration among healthcare team members to tailor treatment plans to individual patient needs.
9. Develop guidelines for managing delirium in various clinical settings, including hospitals, long-term care facilities, and the community.
10. Educate healthcare providers on the principles of titration and de-escalation when using medications to manage delirium symptoms.
11. Prioritise delirium treatment as an essential component of interdisciplinary care plans.
12. Highlight the importance of involving patients and families in treatment decisions and providing them with appropriate education.
13. Foster a patient-centred approach to delirium treatment that respects patient preferences and values.
14. Educate healthcare providers on the ethical considerations of delirium treatment, including informed consent and advanced care planning.
15. Promote ongoing research and evidence dissemination in the field of delirium treatment.

**Prevention and Management**

1. Develop strategies for delirium prevention in various settings, emphasising patient safety and healthcare quality.
2. Create comprehensive care plans for managing delirium, including non-pharmacological approaches tailored to individual patient needs.
3. Implement non-pharmacological interventions effectively, such as sensory aids, orientation techniques, and psychological support.
4. Determine pharmacological interventions for delirium management, considering individual patient factors, adverse effects, and monitoring.
5. Identify best practices for interdisciplinary delirium care and communication among healthcare professionals.
6. Evaluate the effectiveness of delirium prevention strategies in diverse healthcare contexts, fostering continuous improvement.
7. Manage delirium in the context of end-of-life care, addressing family and patient preferences with sensitivity.
8. Consider cultural competence in delirium management, respecting diverse cultural beliefs and practices.
9. Promote interdisciplinary teamwork in delirium prevention, recognition, and management, understanding the roles of each healthcare professional.
10. Collaborate with other healthcare professionals to assess, prevent, and manage delirium effectively, recognising their unique contributions and expertise.
11. Engage in health promotion activities to educate the public, patients, and caregivers about delirium prevention and early recognition.
12. Explore the impact of interdisciplinary teamwork in delirium care on healthcare outcomes and patient satisfaction.

**Types of Delirium**

1. Differentiate between hyperactive, hypoactive, and mixed types of delirium, emphasising their unique characteristics.
2. Understand the clinical implications and management strategies for hyperactive delirium, including patient agitation.
3. Recognise the characteristics and challenges associated with hypoactive delirium, focusing on early detection.
4. Manage mixed delirium, considering the rapid shifts in symptoms and tailoring interventions accordingly.

**Delirium and Dementia**

1. Differentiate between delirium and dementia in terms of onset and attention levels, highlighting distinct features.
2. Recognise that delirium can occur alongside dementia but has distinct characteristics and communicate this to patients and families.
3. Prioritise delirium superimposed on dementia (DSD) in healthcare education, understanding the unique challenges it presents.
4. Recognise DSD as a critical area of study to improve diagnostic accuracy and develop tailored interventions.
5. Differentiate between delirium, dementia, and DSD in educational curricula, highlighting their distinct features.
6. Emphasise the importance of early recognition and differentiation of DSD, as it impacts treatment and outcomes.
7. Prioritise strategies for assessing cognitive function in patients with dementia and coexisting delirium.
8. Develop educational content focusing on risk factors that predispose individuals with dementia to delirium.
9. Address the ethical considerations in the management of DSD, balancing patient autonomy and best interests.
10. Explore pharmacological and non-pharmacological treatment options specific to DSD, considering patient preferences and risks.
11. Engage healthcare professionals in discussions about the challenges and complexities of DSD care, promoting interdisciplinary collaboration.
12. Prioritise research and evidence-based practices in DSD management, aligning education with evolving knowledge.
13. Emphasise the importance of family and caregiver education in recognising and managing DSD.
14. Advocate for increased awareness of DSD among healthcare providers, fostering a proactive approach to diagnosis and treatment.

**Impact on Patients and Families**

1. Assess the psychological impact of delirium on patients and their families, providing comprehensive support.
2. Provide ongoing education to families and caregivers affected by delirium, emphasising their role in patient care.
3. Address the emotional impact of delirium, including anxiety and depression, through interdisciplinary interventions.
4. Recognise ethical and legal considerations in delirium care, especially in vulnerable populations, and advocate for patient rights.
5. Engage with families to support those affected by delirium, involving them in care decisions and offering emotional support.
6. Promote patient-centred care in delirium management, respecting individual preferences and fostering dignity.
7. Understand the economic implications of delirium, including healthcare costs and resource allocation, and advocate for efficient resource use.
8. Foster interdisciplinary research on the holistic impact of delirium, considering patient and family perspectives.

**Health Promotion and Public Education**

1. Prioritise health promotion and public education campaigns focused on delirium prevention and recognition.
2. Develop strategies to educate the public about delirium, its risk factors, and early signs.
3. Engage community organisations and support networks in raising awareness about delirium among at-risk populations.
4. Promote the importance of regular cognitive assessments and delirium risk reduction strategies within the community.
5. Collaborate with schools and educational institutions to include delirium awareness in curricula for healthcare careers.
6. Involve community leaders and influencers in spreading awareness about delirium and its impact.
7. Prioritise family and caregiver education on delirium recognition, prevention, and management.
8. Develop user-friendly educational materials and resources for patients, families, and the community.
9. Advocate for government and healthcare policies that support delirium education and awareness initiatives.
10. Explore innovative methods for disseminating delirium education and resources, including digital platforms and social media.

**Learning Preferences**

1. Learning about delirium through online resources and blended approaches.
2. Learning about delirium through hands-on clinical experiences for practical skill development in delirium assessment and management.
3. Have access to accredited online resources and e-learning modules with interactive features.
4. Education that integrates technology in delirium education, such as virtual simulations and telehealth applications.
5. Module exams or assessments specific to delirium knowledge and skills, focusing on competency-based evaluations.
6. Learning through complex case-based discussions and simulation-based training.
7. Learning with or alongside interdisciplinary colleagues in delirium care.

**Curriculum Integration**

1. Incorporate delirium education into undergraduate healthcare programmes, addressing different healthcare disciplines, including nursing, medicine, and pharmacy.
2. Align delirium education with national guidelines and standards for healthcare education, ensuring consistency and quality.
3. Develop specialised delirium education modules for specific populations, including paediatrics, geriatrics, and intensive care settings.
4. Explore opportunities for interprofessional delirium education, fostering collaboration among healthcare disciplines.
5. Foster lifelong learning and continuous professional development in delirium care, considering evolving best practices and research findings.
6. Incorporate delirium awareness in healthcare curriculum beyond the undergraduate level, including postgraduate and continuing education.
7. Consider the integration of delirium education in postgraduate training for healthcare professionals, promoting advanced expertise.

**Supplementary material 2 - Delphi Survey Items**

| Item | Round 1 (72 items) | Round 2 (31 items) |
| --- | --- | --- |
| Assessment and diagnosis | | |
| 1 | Recognition of the clinical signs and symptoms of delirium |  |
| 2 | Differentiation between hyperactive, hypoactive, and mixed types of delirium |  |
| 3 | Differentiation between delirium and dementia |  |
| 4 | Identification of delirium across different populations | Included |
| 5 | Differentiation between delirium and other cognitive disorders | Included |
| 6 | Recording comprehensive history alongside clinical examination |  |
| 7 | Evaluation of delirium fluctuation in symptoms to tailor interventions |  |
| 8 | Application of standardised delirium assessment tools |  |
| 9 | Assessment of delirium risk factors |  |
| 10 | Interpretation of laboratory and imaging findings relevant to delirium diagnosis | Included |
| 11 | Recognition of delirium in people with comorbid conditions | Included |
| 12 | Assessment of delirium in people with communication challenges |  |
| 13 | Consideration of cultural factors when assessing delirium | Included |
| 14 | Education of people, families, and caregivers about recognising early signs of delirium |  |
| Aetiology and risk factors | | |
| 15 | Identification of common risk factors for delirium |  |
| 16 | Understanding the importance of pain in delirium development |  |
| 17 | Recognition of perioperative care as a risk factor in delirium development |  |
| 18 | Recognition of medications associated with delirium risk |  |
| 19 | Analysis of environmental factors influencing delirium development |  |
| 20 | Understanding of the role of infection in delirium |  |
| 21 | Assessment of the impact of pre-existing cognitive impairment on delirium risk | Included |
| 22 | Recognition of the role of sleep disturbances in delirium | Included |
| Pathophysiology | | |
| 23 | Awareness of the underlying pathophysiology of delirium | Included |
| 24 | Understanding of the neurochemical imbalances involved in delirium | Included |
| 25 | Understanding neurodegenerative processes contributing to delirium | Included |
| 26 | Recognition of how delirium accelerates cognitive decline | Included |
| Treatment of delirium | | |
| 27 | Promotion of effective communication and reorientation strategies | Included |
| 28 | Highlighting the role of non-pharmacological interventions in delirium treatment |  |
| 29 | Highlighting the role of pharmacological interventions for delirium treatment |  |
| 30 | Education on the judicious use of antipsychotic medications in managing delirium |  |
| 31 | Promotion of the psychological interventions in the treatment of delirium |  |
| 32 | Re-evaluation of delirium interventions | Included |
| 33 | Multidisciplinary team collaboration in tailoring treatment and intervention plans |  |
| 34 | Using evidence-based guidelines for management of delirium appropriate to clinical setting |  |
| 35 | Education on the principles of titration in delirium medication management |  |
| 36 | De-escalation and support for persons in distress |  |
| 37 | Involving the person and caregivers in treatment decisions |  |
| 38 | Fostering a person-centred approach to delirium treatment |  |
| 39 | Education on ethical considerations of delirium management | Included |
| Prevention | | |
| 40 | Developing strategies for delirium prevention in various settings | Included |
| 41 | Creating anticipatory care plans to minimise delirium occurrence |  |
| 42 | Minimising the impact of environmental changes in the prevention of delirium |  |
| 43 | Awareness of the importance of reorientation for people at higher risk of delirium |  |
| 44 | Minimisation of physiological risk factors through pharmacological interventions to reduce delirium risk |  |
| 45 | Highlighting the importance of regular medication review in delirium prevention |  |
| 46 | Promotion of evidence-based communication strategies to minimise delirium occurrence |  |
| 47 | Tailoring delirium prevention strategies for diverse healthcare settings | Included |
| 48 | Tailoring delirium prevention strategies for diverse cultures | Included |
| 49 | Promoting multidisciplinary care in dementia prevention |  |
| Delirium and dementia | | |
| 50 | Understanding that delirium can occur alongside dementia |  |
| 51 | Awareness of delirium superimposed on dementia (DSD) |  |
| 52 | Emphasising the importance of early recognition and differentiation of DSD | Included |
| 53 | Prioritising strategies for assessing cognitive function in people with delirium and coexisting dementia | Included |
| 54 | Highlighting risk factors that predispose individuals with dementia to delirium |  |
| 55 | Addressing ethical considerations in the management of DSD | Included |
| 56 | Exploring pharmacological and non-pharmacological treatment options specific to DSD | Included |
| Impact on people with delirium, informal caregivers and family | | |
| 57 | Assessing the psychological impact of delirium on the person, their caregivers and family | Included |
| 58 | Providing ongoing education to families and caregivers affected by delirium |  |
| 59 | Addressing the emotional impact of delirium |  |
| 60 | Recognising legal considerations in delirium care | Included |
| 61 | Proactive communication with caregivers and families to help them support those affected by delirium |  |
| 62 | Promoting person-centred care in delirium management |  |
| 63 | Understanding the economic implications of delirium and advocating for efficient resource use | Included |
| Health promotion and public education | | |
| 64 | Prioritising health promotion and public education focused on delirium prevention and recognition | Included |
| 65 | Developing strategies to educate the public about delirium | Included |
| 66 | Engaging community organisations and support networks in raising awareness about delirium among at-risk populations | Included |
| 67 | Promoting the importance of regular cognitive assessments and delirium risk reduction strategies within the community | Included |
| 68 | Collaborating with further and higher education institutions to include delirium awareness in curricula for healthcare careers | Included |
| 69 | Prioritising family and caregiver education on delirium |  |
| 70 | Developing user-friendly educational materials for the public |  |
| 71 | Advocating for government and healthcare policies that support delirium education and awareness initiatives | Included |
| 72 | Exploring innovative methods for disseminating delirium education | Included |

**Supplementary material 3 – Percentage consensus “in” after two Delphi rounds**

*Those shaded in green met the 75% threshold and were therefore included in the final list of educational priorities.*

| Item | | Round 1 | Round 2 |
| --- | --- | --- | --- |
| Assessment and diagnosis | | | |
| 1 | Recognition of the clinical signs and symptoms of delirium | 93.75% | N/A |
| 2 | Differentiation between hyperactive, hypoactive, and mixed types of delirium | 95% | N/A |
| 3 | Differentiation between delirium and dementia | 91.25% | N/A |
| 4 | Identification of delirium across different populations | 42.50% | 18.18% |
| 5 | Differentiation between delirium and other cognitive disorders | 66.25% | 49.09% |
| 6 | Recording comprehensive history alongside clinical examination | 97.50% | N/A |
| 7 | Evaluation of delirium fluctuation in symptoms to tailor interventions | 85% | N/A |
| 8 | Application of standardised delirium assessment tools | 92.50% | N/A |
| 9 | Assessment of delirium risk factors | 100% | N/A |
| 10 | Interpretation of laboratory and imaging findings relevant to delirium diagnosis | 58.75% | 12.73% |
| 11 | Recognition of delirium in people with comorbid conditions | 70% | 50.91% |
| 12 | Assessment of delirium in people with communication challenges | 97.50% | N/A |
| 13 | Consideration of cultural factors when assessing delirium | 26.25% | 40% |
| 14 | Education of people, families, and caregivers about recognising early signs of delirium | 97.50% | N/A |
| Aetiology and risk factors | | | |
| 15 | Identification of common risk factors for delirium | 98.75% | N/A |
| 16 | Understanding the importance of pain in delirium development | 96.25% | N/A |
| 17 | Recognition of perioperative care as a risk factor in delirium development | 86.25% | N/A |
| 18 | Recognition of medications associated with delirium risk | 91.25% | N/A |
| 19 | Analysis of environmental factors influencing delirium development | 100% | N/A |
| 20 | Understanding of the role of infection in delirium | 98.75% | N/A |
| 21 | Assessment of the impact of pre-existing cognitive impairment on delirium risk | 75% | 52.73% |
| 22 | Recognition of the role of sleep disturbances in delirium | 62.50% | 58.18% |
| Pathophysiology | | | |
| 23 | Awareness of the underlying pathophysiology of delirium | 48.75% | 90.91% |
| 24 | Understanding of the neurochemical imbalances involved in delirium | 28.75% | 27.27% |
| 25 | Understanding neurodegenerative processes contributing to delirium | 45% | 40% |
| 26 | Recognition of how delirium accelerates cognitive decline | 70% | 72.73% |
| Treatment of delirium | | | |
| 27 | Promotion of effective communication and reorientation strategies | 100% | N/A |
| 28 | Highlighting the role of non-pharmacological interventions in delirium treatment | 100% | N/A |
| 29 | Highlighting the role of pharmacological interventions for delirium treatment | 86.25% | N/A |
| 30 | Education on the judicious use of antipsychotic medications in managing delirium | 88.75% | N/A |
| 31 | Promotion of the psychological interventions in the treatment of delirium | 72.50% | 61.82% |
| 32 | Re-evaluation of delirium interventions | 50% | 58.18% |
| 33 | Multidisciplinary team collaboration in tailoring treatment and intervention plans | 90% | N/A |
| 34 | Using evidence-based guidelines for management of delirium appropriate to clinical setting | 97.5% | N/A |
| 35 | Education on the principles of titration in delirium medication management | 78.75% | N/A |
| 36 | De-escalation and support for persons in distress | 96.25% | N/A |
| 37 | Involving the person and caregivers in treatment decisions | 100% | N/A |
| 38 | Fostering a person-centred approach to delirium treatment | 98.75% | N/A |
| 39 | Education on ethical considerations of delirium management | 53.75% | 74.55% |
| Prevention | | | |
| 40 | Developing strategies for delirium prevention in various settings | 73.75% | 76.36% |
| 41 | Creating anticipatory care plans to minimise delirium occurrence | 78.75% | N/A |
| 42 | Minimising the impact of environmental changes in the prevention of delirium | 93.75% | N/A |
| 43 | Awareness of the importance of reorientation for people at higher risk of delirium | 95% | N/A |
| 44 | Minimisation of physiological risk factors through pharmacological interventions to reduce delirium risk | 82.50% | N/A |
| 45 | Highlighting the importance of regular medication review in delirium prevention | 98.75% | N/A |
| 46 | Promotion of evidence-based communication strategies to minimise delirium occurrence | 83.75% | N/A |
| 47 | Tailoring delirium prevention strategies for diverse healthcare settings | 75% | 36.36% |
| 48 | Tailoring delirium prevention strategies for diverse cultures | 63.75% | 25.45% |
| 49 | Promoting multidisciplinary care in dementia prevention | 95% | N/A |
| Delirium and dementia | | | |
| 50 | Understanding that delirium can occur alongside dementia | 92.5% | N/A |
| 51 | Awareness of delirium superimposed on dementia (DSD) | 83.75% | N/A |
| 52 | Emphasising the importance of early recognition and differentiation of DSD | 75% | 60% |
| 53 | Prioritising strategies for assessing cognitive function in people with delirium and coexisting dementia | 66.25% | 50.91% |
| 54 | Highlighting risk factors that predispose individuals with dementia to delirium | 85% | N/A |
| 55 | Addressing ethical considerations in the management of DSD | 61.25% | 21.82% |
| 56 | Exploring pharmacological and non-pharmacological treatment options specific to DSD | 66.25% | 70.91% |
| Impact on people with delirium, informal caregivers and family | | | |
| 57 | Assessing the psychological impact of delirium on the person, their caregivers and family | 70% | 94.55% |
| 58 | Providing ongoing education to families and caregivers affected by delirium | 92.50% | N/A |
| 59 | Addressing the emotional impact of delirium | 87.50% | N/A |
| 60 | Recognising legal considerations in delirium care | 63.75% | 72.73% |
| 61 | Proactive communication with caregivers and families to help them support those affected by delirium | 87.50% | N/A |
| 62 | Promoting person-centred care in delirium management | 97.50% | N/A |
| 63 | Understanding the economic implications of delirium and advocating for efficient resource use | 53.75% | 54.55% |
| Health promotion and public education | | | |
| 64 | Prioritising health promotion and public education focused on delirium prevention and recognition | 73.75% | 83.64% |
| 65 | Developing strategies to educate the public about delirium | 61.25% | 47.27% |
| 66 | Engaging community organisations and support networks in raising awareness about delirium among at-risk populations | 43.75% | 56.36% |
| 67 | Promoting the importance of regular cognitive assessments and delirium risk reduction strategies within the community | 43.75% | 38.18% |
| 68 | Collaborating with further and higher education institutions to include delirium awareness in curricula for healthcare careers | 72.50% | 23.64% |
| 69 | Prioritising family and caregiver education on delirium | 91.25% | N/A |
| 70 | Developing user-friendly educational materials for the public | 82.5% | N/A |
| 71 | Advocating for government and healthcare policies that support delirium education and awareness initiatives | 56.25% | 43.64% |
| 72 | Exploring innovative methods for disseminating delirium education | 47.50% | 63.64% |

**Supplementary material 4 – DELPHISTAR Reporting Guidelines**

##
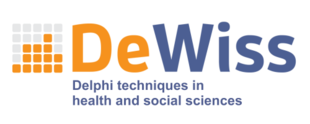

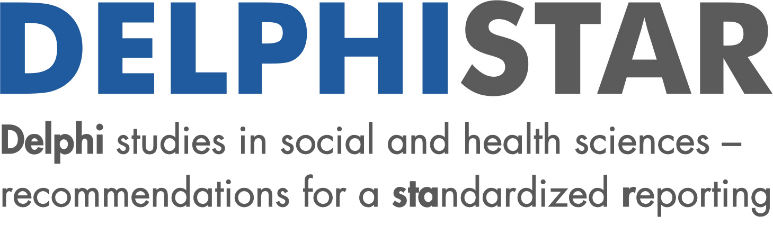

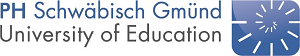


**Delphi studies in social and health sciences – recommendations for an interdisciplinary standardized reporting (DELPHISTAR).**

From: Niederberger, M., Schifano, J., Deckert, S., Hirt, J., Homberg, A., Köberich, S., Kuhn, R., Rommel, A., Sonnberger, M. & the DEWISS network (2024). Delphi studies in social and health sciences—Recommendations for an interdisciplinary standardized reporting (DELPHISTAR). Results of a Delphi study. *PLoS ONE 19(8):* e0304651. <https://doi.org/10.1371/journal.pone.0304651>

More information under OSF (<https://osf.io/gc4jk>) and DEWISS (<https://delphi.ph-gmuend.de/>)

**What is the aim of DELPHISTAR?**

- Improve, harmonize, and make the reporting in publications on Delphi studies comparable
- Facilitate the evaluation of Delphi studies including during peer review processes
- Reduce, and ideally prevent, inconsistencies and unclear descriptions in publications on Delphi studies
- Raise awareness of the diversity among the Delphi variants and of their specific potentials and challenges

**DELPHISTAR is a Delphi reporting guideline that is:**

- valid for all Delphi variants (e.g., classic Delphi, real-time Delphi, group Delphi, policy Delphi, argumentative Delphi, café Delphi)
- applicable to different purposes (e.g., Delphi studies to establish consensus, to gather expert judgments or to forecast)
- given equal consideration in the health and social sciences

**This reporting guideline is meant for studies using Delphi techniques in the health and social sciences.** These also include all Delphi variants and modifications that meet the following criteria:

1. Survey of several people (=experts) with specialized knowledge (e.g., operational knowledge, experiential knowledge, functional knowledge, contextual knowledge)
2. Structured communication process
3. Carrying out at least two survey rounds or the option to respond at least two times
4. Feedback: the (interim) results are presented to the experts starting from the second round
5. Basis is a quantitative questionnaire with the possibility to contribute or supplement arguments for the respective position
6. Quantitative and qualitative answer are systematically analyzed (quantitative: e.g., descriptive statistics, qualitative: e.g., thematic analysis)

This reporting guideline is available in English and German at <https://delphi.ph-gmuend.de/activities/delphistar> (last update October 2024).

**Contact**

Prof. Dr. Marlen Niederberger

E-mail: marlen.niederberger@ph-gmuend.de

Department of Research Methods in Health Promotion and Prevention

Institute for Health Sciences, University of Education Schwäbisch Gmünd,

Oberbettringer Straße 200, 73525 Schwäbisch Gmünd, Germany

| **Topic** | **Section** | **Item** | **Checklist Item** | **Location where item is reported** | **Exemplary wording** |
| --- | --- | --- | --- | --- | --- |
| **I**  **Title and Abstract** |  | 1 | Identification as a Delphi study in the title | Page 1, Title | What is a public health intervention? Results of a Delphi study. |
|  |  | 2 | Identification as a Delphi study in the abstract | Page 2, Abstract | A Delphi study was selected to answer the research question. |
|  |  | 3 | Structured abstract | Page 2 and 3, Abstract | e.g., background, method, results and discussion |
| **II**  **Context** | **Formal** | 4 | Information about the sources of funding | Page 22, Funding | The Delphi study was funded by [SOURCE]. |
|  |  | 5 | Information about the team of authors and/or researchers (e.g., discipline, institution) | Page 1 | The Delphi study was conducted by an interdisciplinary team with representatives from medicine, public health, and health promotion. |
|  |  | 6 | Information about method consulting | Page 6/7, Study Design | The study group was advised by experts from [INSTITUTION] regarding statistics.  Or:  No consulting in regard to method took place. |
|  |  | 7 | Information about the project background | Page 5/6, Background | The Delphi study was part of a mixed-methods study on [AIM]. |
|  |  | 8 | Information about the study protocol | Page 5/6, Background | The study protocol is available at [LINK]. |
|  | **Content** | 9 | Justification of the chosen method (Delphi) to answer the research question | Page 5/6, Background | The Delphi method is suitable for answering the research question because it systematically gathers the judgments of different expert groups and can identity agreement and disagreement. |
|  |  | 10 | Aim of the Delphi study (e.g., consensus, forecasting) | Page 5/6, Background | The aim of the Delphi study is to find consensus on criteria to define a public health intervention. |
| **III**  **Method** | **Body & Integration of knowledge** | 11 | Identification and elucidation of relevant expertise, spheres of experience, and perspectives (e.g., theory, practice, affected groups, disciplines) | Page 7, Recruitment/ population | The experts represent the sciences and clinical practice because [REASON]. |
|  |  | 12 | Handling of knowledge, expertise and perspectives which are missing or have been deliberately not integrated | Page 7, Recruitment/ population | If it is not possible to recruit experts specialized in [AREA], this will be openly communicated to the other experts during the Delphi study. |
|  |  | 13 | Basic definition of expert^1^ | Page 7, Recruitment/ population | A person who has been active in the area for at least [NUMBER] years is considered to be an expert. |
|  | **Delphi variant and modifications** | 14 | Identification of the type of Delphi variant and potential modifications (e.g., classic Delphi, real-time Delphi, group Delphi) | Page 6/7, study design | A classic Delphi study was used [LITERATURE REFERENCE]. |
|  |  | 15 | Justification of the Delphi variant and modifications, including during the Delphi study, if applicable | Page 6/7, study design | If the willingness to participate clearly decreases between the first and second round, a third round will not be held. |
|  | **Sample of experts** | 16 | Selection criteria for the experts (per round, per expert group if applicable) | Page 7, Recruitment/ population | All of the experts who met the definition were invited to the first round.  All of the experts who completed the previous round were invited to participate in the subsequent round. |
|  |  | 17 | Identification of the experts | Page 7, Recruitment/ population | The experts were identified based on publications in [DATABASE]. |
|  |  | 18 | Information about recruiting and any subsequent recruiting of experts | Page 7, Recruitment/ population | The experts were informed about the Delphi study and invited to participate. |
|  | **Survey** | 19 | Elucidation of the content development for the questionnaire^2^ | Page 8/9, Development of the Delphi Survey | The questionnaire was developed based on the results of systematic reviews [LITERATURE REFERENCE]. |
|  |  | 20 | Description of the questionnaire (content and structure) | Page 8/9, Development of the Delphi Survey | The questionnaire was divided into three segments on [TOPICS]. The statements made in the questionnaire were evaluated using standardized items, with the option to comment in free-text boxes. |
|  | **Delphi rounds** | 21 | Number of Delphi rounds | Page 6/7, Study Design | Three Delphi rounds were held. |
|  |  | 22 | Information about the aims of the individual Delphi rounds | Page 9/10, First round and Second round | The first Delphi round focused on exploring relevant aspects. These aspects were then presented to the experts in the second Delphi round for standardized evaluation. |
|  |  | 23 | Disclosure and justification of the criterion for discontinuation | Page 6/7, Study Design | The number of rounds was defined in advance to be a maximum of three rounds. |
|  | **Feedback** | 24 | Information about what data was reported back per round | Page 9/10, Second round | In terms of feedback, we shared the statistical results plus the summary of the open responses. |
|  |  | 25 | Information on how the results of the previous Delphi round were fed back to the experts surveyed (e.g., via frequencies, mean values, measures of dispersion, listing of comments) | Page 9/10, Second round | Mean values, standard deviations and percentage frequency distributions were reported. |
|  |  | 26 | Information on whether feedback was differentiated by specific groups (e.g., by field of expertise, institutional affiliation) | Page 11, Analysis | The feedback was aggregated across all expert groups. |
|  |  | 27 | Information about how dissent and unclear results were handled | Page 9/10, Second round | The results showing dissent were presented again for evaluation in the next Delphi round. |
|  | **Data analysis** | 28 | Disclosure of the quantitative and qualitative analytical strategy | Page 11, Analysis | The quantitative items were descriptively analyzed. The open-ended items were analyzed using thematic analysis [LITERATURE REFERENCE]. |
|  |  | 29 | Definition and measurement of consensus | Page 11, Analysis | Consensus was defined as percentage agreement, meaning that agreement was assumed if at least 80% of the respondents agreed on an item. |
|  |  | 30 | Information on group-specific analysis or weighting of experts (e.g., theory vs. practice, discipline-specific analysis) | Page 11, Analysis | In the analysis, the mean values for percent agreement are weighted for each expert group in terms of the number of group members. |
| **IV**  **Results** | **Delphi process** | 31 | Illustration of the Delphi study (e.g., in a flow chart) | Page 9/10, Second Round | A summary of the Delphi study is illustrated in a flow chart (Figure 1). |
|  |  | 32 | Information about special aspects during the Delphi study (e.g., deviations from the intended approach with justification) | Page 6/7, Study Design | During the Delphi study the political discussion mentioned climate change and the effects on health. It is possible that this influenced the experts' responses. |
|  |  | 33 | Number of experts per round (both invited and participating) | Page 11/12, Results | The number of experts participating in the first Delphi round was [NUMBER], and the number of experts in the second round was [NUMBER]. This corresponds to a response rate of [NUMBER]% in the first round and [NUMBER]% in the second round. |
|  | **Results** | 34 | Presentation of the results for each Delphi round and the final results | Page 12/ 13/ 14, Results | In the first Delphi round [NUMBER]% of the experts agreed, in the second [NUMBER]%, and in the third [NUMBER]%. |
| **V Discussion** | **Quality of findings** | 35 | Highlighting the findings from the Delphi study | Page 17, Discussion | The central findings can be summarized as follows: [STATE FINDINGS]. |
|  |  | 36 | Validity of the results (e.g., transferability of the findings) | Pages 19/20/21, Strengths and Limitations | The results are not transferable to other countries due to different legal regulations. |
|  |  | 37 | Reliability of the results (e.g., split half, inter-rater reliability) | Pages 19/20/21, Strengths and Limitations | The responses in the free-text comments were analyzed by two independent reviewers [SPECIFY]. |
|  |  | 38 | Reflection on potential limitations (e.g., number of experts, response bias) | Page 14 and 15, Strengths and Limitations | The results are to be viewed critically with regard to the composition of the panel because [REASONS]. |

^1^ “Experts” are the participants; these can be people from academia, practice, or representatives of lived experience (e.g., patients, family members).

^2^ The term “questionnaire” stands for the survey instrument regardless of whether quantitative or qualitative items are integrated or weighted.
